# Examining the experience of childbirth and its predictors among women who have recently given birth

**DOI:** 10.1002/nop2.603

**Published:** 2020-08-19

**Authors:** Faeze Khalife‐Ghaderi, Leila Amiri‐Farahani, Shima Haghani, Syedeh Batool Hasanpoor‐Azghady

**Affiliations:** ^1^ Department of Reproductive Health and Midwifery School of Nursing and Midwifery Iran University of Medical Sciences Tehran Iran; ^2^ Department of Reproductive Health and Midwifery Nursing Care Research Center School of Nursing and Midwifery Iran University of Medical Sciences Tehran Iran; ^3^ Department of Biostatistics Nursing Care Research Center Iran University of Medical Sciences Tehran Iran

**Keywords:** childbirth experience, pregnancy experience, presence of a companion, satisfaction with the birth environment

## Abstract

**Aims:**

The aim of this study is to examine the experience of childbirth and its predictors among women who have recently given birth.

**Design:**

This is a cross‐sectional study.

**Methods:**

This study was conducted on 225 women at 22 Bahman Hospital in Khaf City, Iran. The samples were selected by the continuous sampling method from August to November 2018. Data were collected by demographic questionnaire, fertility information, pregnancy experience scale, satisfaction from birth environment inventory and the childbirth experience questionnaire.

**Results:**

The mean score of childbirth experience was 55.73. According to the regression model, the husband's education, receiving regular care during pregnancy, the person giving birth, presence of a companion, receiving spinal anaesthesia, perineal conditions, being uplifted and hassled about the pregnancy and satisfaction with the birth environment were the predictors of childbirth experience. The regression model showed 39.8% of the change in outcome variable was predicted by independent variables.

## 1 INTRODUCTION

Pregnancy, delivery and their experiences are among natural processes. They are also exciting and important events in the life of any woman and family (Toohill et al., 2014). Childbirth experience is one of the factors that affect the quality of life of women and having negative experiences in this regard can have immediate and long term adverse effects on the general health of mothers and their families (Larkin, Begley, & Devane, 2012). Women, who are not satisfied with their childbirth process, associate their childbirth with pain, anger, fear and sadness (Fair & Morrison, 2012) and having undesirable experience of childbirth can lead to postpartum depression or post‐traumatic stress disorder (PTSD), (Fenwick, Staff, Gamble, Creedy, & Bayes, 2010). The negative experience of childbirth can lead to the inability to have sex and tendency toward the caesarean section (McLachlan et al., 2012).

According to the studies conducted in this area, some factors that affect the experience of childbirth include; childbirth experience, the environment of labour and delivery room (Larsson, Saltvedt, Edman, Wiklund, & Andolf, 2011), demographic characteristics and the individual's expectations that match the childbirth experience (Fair & Morrison, 2012). Some factors such as childbirth preparation classes, receiving information about childbirth, mental support and especially, the childbirth relaxation classes influence the self‐confidence and courage of women in labour (Nasiry & Sharifi, 2013). Also, we can refer to the experiences that are gained during pregnancy, which affect the childbirth experience (Lobel et al., 2008). Feeling the foetus's movement is an exciting and enjoyable experience for all women that often results in women's content and greater adaptation to the stress of pregnancy (Babanazari & Kafi, 2007). However, approaching labour time is stressful for most women and may lead to negative experiences of childbirth in them (Avni‐Barron & Wiegartz, 2011).

According to similar studies, the appropriate environment is considered as one of the most important factors that affect the childbirth process. Providing a specific environment for every woman in labour to enjoy a quiet place without noise and with mild colours and respecting the privacy of patients can have a considerable role in the satisfaction of mothers and desirable experience of childbirth (Oyira et al., 2016). Providing a secure and appropriate environment for childbirth, support of women by midwifery personnel, giving the real explanation of childbirth process in a simple language and avoiding unnecessary interventions that are provided to all women regardless of their needs can be effective in creating a positive experience of childbirth (Askari, Atarodi, Torabi, & Moshki, 2014).

The studies and researches conducted across the world mostly indicate the negative perception of women toward childbirth (Elvander, Cnattingius, & Kjerulff, 2013), although, in some studies, women were satisfied with their childbirth and considered it a positive experience (Bell & Andersson, 2016). If women do not have proper knowledge and perception about their childbirth experiences, their improper perception may pass on to other women and negatively affect them (Dolatian, Sayyahi, & Simbar, 2008).

Although childbirth is considered a natural process and a unique phenomenon in the universe that can be tolerated by most women, identification of factors that create the positive or negative experience of natural childbirth is one of the most fundamental steps toward propagating natural childbirth and decreasing caesarean sections.

There are limited and contradictory data regarding the experience of childbirth and its predictors among Iranian women. Hence, the present study was designed to determine the childbirth experience and its predictors among women who have recently given birth.

## 2 METHODS

### 2.1 Design and sample

This is a cross‐sectional study that was conducted on women who had recently given birth at 22 Bahman Hospital in Khaf County in Razavi Khorasan Province from August to November 2018. The sample size was estimated to be 225 samples with $z_{1‐\frac{\alpha }{2}}=1.96$, W = 0.05 (assuming the coefficient of determining all factors connected with the childbirth experience of the women as outcome variable), *R* ^2^ = 0.2 (what part of the outcome variable justifies the influential variables?), $R_{x,x_{j}}^{2}=0.7$ (correlation between the main influential variable and other influential variables) and *p* = 20 (number of influential variables) in the following formula.

$$n={\frac{\left(z_{1}‐\frac{\alpha }{2}\right)}{w}}^{2}\cdot \left(\frac{1‐R^{2}}{1‐R_{x,x_{j}}^{2}}\right)+P+1.$$

The sampling was carried out by the continuous method. The inclusion criteria were; being 15–45 years old woman with reading and writing ability, being Iranian, having natural childbirth in the current pregnancy, having a full‐term infant who is healthy according to the physical examination after delivery, being in the latent phase or at the beginning of active phase (3–4 cm) and having a singleton pregnancy. The exclusion criteria were; having any special illness or conditions that make pregnancy high risk, having stressful experiences in the past 3 months, requiring serious midwifery interventions after birth and the need for infant resuscitation immediately after delivery and transfer to the neonatal intensive care unit.

### 2.2 Instruments/outcome and influential variables

The data were collected using a four‐part demographic and fertility information form, pregnancy experience scale, satisfaction with the birth environment inventory and childbirth experience questionnaire.

Dencker's childbirth experience questionnaire (CEQ) was also used to assess childbirth experience as an outcome variable. The CEQ designed by Denker includes 21 items and 4 domains, including own capacity (8 items), perceived safety (6 items), professional support (4 items) and participation (3 items). For items 1–18, the 4‐point Likert scale was used that ranged from totally agree (score 4), mostly agree (3), mostly disagree (2) and totally disagree (1). The total score of childbirth experience ranged from 21 to 84. The validity of CEQ was confirmed by Cronbach's alpha of ≥0.7 (Dencker, Taft, Bergqvist, Lilja, & Berg, 2010). The reliability of the Persian version of CEQ was confirmed by α = 0.82 (Abbaspoor, Moghaddam‐Banaem, Ronaghi, & Dencker, 2019). The pregnancy experience scale, the birth environment satisfaction questionnaire and demographic and fertility information form were used to identify the influential variables of the childbirth experience.

DiPietro's pregnancy experience scale includes 20 items and 2 domains; including being hassled and uplifted during the pregnancy (10 items focus on the hassle and 10 items focus on uplift). The 4‐point Likert scale is used in this scale that ranges from none (score 0) to high (score 3). The minimum score is 0 and the maximum score is 30 and the higher score indicates greater hassle or uplift for each domain. DiPietro obtained the reliability coefficient of being uplifted to be 0.82 and being hassled to be 0.83 (DiPietro, Ghera, Costigan, & Hawkins, 2004). The reliability of uplifted and hassled was calculated to be 0.77 and 0.69, respectively (Hajifoghaha, Abbas, & Nourossadat, 2016).

The birth environment satisfaction questionnaire includes 17 questions. The samples in this questionnaire rate their satisfaction or dissatisfaction in a 5‐point Likert scale that ranges from very dissatisfied (score 0), dissatisfied (1), no comment (2), satisfied (3) and very satisfied (4). At the end, the scores are added and calculated as percentage. In this study, the subjects were placed in four categories in terms of satisfaction with the birth environment as follows: 0–24 = very dissatisfied, 25–50 = dissatisfied, 51–75 = satisfied and 76–100 = very satisfied. The validity of this questionnaire has been confirmed by the content validity method and its reliability has been confirmed by Cronbach's alpha (a = 0.92) and (a = 0.9) (Dolatian et al., 2008). In the present study, 4 questions of the 17‐item questionnaire designed by Dolatian et al. were removed as the labour was included in LDR (labour/delivery room) and the delivery room was the same.

The demographic and fertility information form consisted of information about Age, husband's age, education level, husband's education level, occupation, husband's occupation, residence, economic status, insurance status, gravidity, parity, Abortion, live children, gestational age, planned pregnancy, getting regular care during pregnancy, place of care during pregnancy, caregiver during pregnancy, participate in the pregnancy class, caregiver during labour, the presence of a companion during labour, the person giving birth, type of delivery, getting epidural or spinal anaesthetic, getting painkiller, perineum status and infant's gender.

### 2.3 Statistical analysis

The statistical analysis was conducted using SPSS software version 21. Descriptive statistics including frequency distribution and also central and disperse indexes such as mean and standard deviation were used to describe childbirth experience and its related factors. Also, independent *t* test and one‐way ANOVA were used to determine the relationship between childbirth experience and demographic and fertility characteristics. Correlation tests were used to determine the relationship between childbirth experience, pregnancy experience and birth environment. Then, all variables that had the value of *p* ˂ .05 entered the multiple linear regression model to estimate the effect of each independent variable (pregnancy experience, birth environment and demographic and fertility variables) on the outcome variable (childbirth experience) and to explain the changes. The significance level of less than 0.05 was considered for statistical tests.

## 3 RESULTS

The mean age of participants was 27.27 years with standard deviation of 5.89. Other demographic and fertility characteristics of participants are presented in Tables 1 and 2.

**Table 1:** Relationship between demographic and fertility characteristics (quantitative variables) and childbirth experience in women (*n* = 225)

Demographic and fertility characteristics		*N* (%)	*p* Value^a^
Age (years)	≤19	18 (8)	*p* = .95^b^
	20–29	131 (58.2)	
	30–39	70 (31.1)	
	≥40	6 (2.7)	
Husband's age (years)	≤29	90 (40)	*p* = .695^b^
	30–39	103 (45.8)	
	≥40	32 (14.2)	
Gravidity	1	62 (27.6)	*p* = .31^b^
	2	60 (26.7)	
	3	45 (20)	
	4	30 (13.3)	
	≥5	28 (12.4)	
Parity	0	67 (29.8)	*p* = .318^b^
	1	69 (30.7)	
	2	50 (22.2)	
	3	20 (9.8)	
	4	19 (8.4)	
Abortion	0	173 (76.9)	*p* = .721^b^
	1	39 (17.33)	
	2	10 (4.44)	
Live children	0	70 (31.1)	*p* = .227^b^
	1	68 (30.2)	
	2	47 (20.9)	
	3	22 (9.8)	
	4	14 (6.2)	
Gestational age	37	11 (4.9)	*p* = .446^b^
	38	47 (20.9)	
	39	74 (32.9)	
	40	79 (35.1)	
	41	14 (6.2)	

^a^ Significance level: *p* < .05.

^b^ One‐way ANOVA test.

**Table 2:** Relationship betwseen demographic and fertility characteristics (categorical variables) and birth experience in women (*n* = 225)

Demographic and fertility characteristics		*N* (%)	*p* Value^a^
Education level	Elementary	49 (21.8)	***p* = .028** b
	Guidance	48 (21.33)	
	High school	75 (33.33)	
	Academic	52 (23.1)	
Occupation	Housewife	187 (83.1)	*p* = .296^c^
	Employee	38 (16.9)	
Husband's education level	Elementary	39 (17.33)	***p* = .009** b
	Guidance	47 (20.88)	
	High school	80 (35.55)	
	Academic	58 (25.8)	
Husband's occupation	Unemployed	6 (2.7)	*p* = .715^b^
	Manual worker	88 (39.11)	
	Employee	41 (18.22)	
	Self‐employed	75 (33.33)	
	Animal husbandry	5 (2.2)	
	Farmer	10 (4.44)	
Residence	Urban	124 (55.1)	*p* = .141^c^
	Rural	101 (44.9)	
Economic status	Rich	13 (5.8)	*p* = .412^b^
	Appropriate	108 (48)	
	Relatively appropriate	94 (41.8)	
	Inappropriate	10 (4.4)	
Insurance status	Yes	191 (84.9)	*p* = .506^c^
	No	34 (15.1)	
Planned pregnancy	Yes	193 (85.8)	*p* = .16^c^
	No	32 (14.2)	
Getting regular care during pregnancy	Yes	216 (96)	***p* = .013** c
	No	9 (4)	
Place of care during pregnancy	Personal office	24 (10.7)	*p* = .697^b^
	Health Center	92 (40.9)	
	Health home	109 (48.4)	
Caregiver during pregnancy	Midwife	107 (47.8)	*p* = .507^b^
	Gynaecologist	20 (8.9)	
	Behvarz^c^	35 (15.66)	
	Health care provider	59 (26.3)	
Participate in the pregnancy class	Yes	33 (14.7)	*p* = .163^c^
	No	192 (85.3)	
The person giving birth	Gynaecologist	12 (5.3)	***p* = .042** b
	Midwife	184 (81.8)	
	Both	29 (12.9)	
Infant's gender	Male	111 (49.3)	*p* = .918^c^
	Female	114 (50.7)	
The presence of a companion during labour	Yes	128 (56.9)	***p* = .047** c
	No	97 (43.1)	
Caregiver during labour	Gynaecologist	5 (2.2)	*p* = .833^b^
	Midwife	188 (83.6)	
	Midwife and Gynaecologist	32 14.2)	
Type of delivery	Physiologic	170 (75.6)	***p* = .047** c
	Intervention	55 (24.4)	
Getting epidural or spinal anaesthetic	Yes	14 (6.2)	***p* = .02** c
	No	211 (93.8)	
Getting painkiller	Yes	56 (24.9)	*p* = .793^c^
	No	169 (75.1)	
Perineum status	No laceration	136 (60.4)	***p* = .03** b
	Episiotomy	20 (8.9)	
	First or second degree tear	69 (30.7)	

^a^ Significance level: *p* < .05 and the bold *p* values consider significant.

^b^ One‐way ANOVA test.

^c^ Independent sample *t* test.

^d^ Behvarz or Community health worker (CHW) are members of a community who are chosen by community members or organizations to provide basic health and medical care to their community capable of providing preventive, promotional and rehabilitation care to these communities.

The mean score of childbirth experience was 55.73 of 100 with standard deviation of 6.98 that was higher than the median score of the tool (52.5). Among the domains of the childbirth experience, professional support with a mean of 3.44 and participation with a mean of 2.04 received the highest and lowest scores, respectively (Table 3).

**Table 3:** Numerical indexes of childbirth experience and its domains in women (*n* = 225)

Birth experience and its domains	Minimum	Maximum	Mean	Standard deviation	Base 1–4			
					Minimum	Maximum	Mean	Standard deviation
Own capacity (8–32)	14	30	21.24	3.47	1.75	3.75	2.65	0.43
Professional support (4–16)	4	16	13.76	2.75	1	4	3.44	0.68
Perceived safety (6–24)	8	23	14.58	2.68	1.33	3.83	2.43	0.44
Participation (3–12)	3	12	6.13	1.77	1	4	2.04	0.59
Childbirth experience (21–84)	35	71	55.73	6.98	1.67	3.38	2.65	0.33

According to the results of this study, there was a significant relationship between the variables of being uplifted and hassled and the domains of childbirth experience (*p* ˂ .001). The satisfaction with the birth environment had a significant correlation with childbirth experience so that the score of childbirth experience increased along with the increase in the satisfaction with the birth environment (*p* ˂ .001), (Table 4).

**Table 4:** Correlation between the birth experience and its domains, experience of pregnancy and its domains and the birth environment in women (*n* = 225)

Childbirth experience and its domains	Pregnancy experience and its domains		Satisfaction with the birth environment
	Uplift	Hassle	
Own capacity	*r* = .273 *p* < .001	*r* = −.199 *p* = .003	*r* = .035 *p* = .601
Professional support	*r* = .204 *p* = .002	*r* = −0.122 *p* = .067	*r* = .546 ***p*** **< .001**
Perceived safety	*r* = .141 *p* = .035	*r* = −.306 *p* < .001	*r* = .103 *p* = .124
Participation	*r* = .138 *p* = .039	*r* = .001 *p* = .998	*r* = .181 ***p*** **= .006**
Childbirth experience	*r* = .305 *p* < .001	*r* = −.265 *p* < .001	*r* = .318 ***p*** **< .001**

Note

Significance level: *p* < .05 and the bold *p* values consider significant.

To estimate the effect of each variable of demographic and fertility characteristics, pregnancy experience and satisfaction with the birth environment on childbirth experience and to explain the changes, all variables with the value of *p* < .05 (based on the results of Tables 1, 2&4) were entered into the multiple linear regression model with the “enter” method. Among the variables entered in the model, the variables of husband's education, receiving regular pregnancy care, the person giving birth, presence of a companion, receiving epidural or spinal anaesthesia, perineal conditions, being uplifted and hassled about pregnancy and satisfaction with the birth environment remained in the model. It should be mentioned that a significance level of *p* ˂ .05 was considered in the present model. According to the results of the study, 39.8% of the changes in the variable of childbirth experience could be explained by independent variables of husband's education, receiving regular care during pregnancy, the person giving birth, presence of a companion, receiving epidural or spinal anaesthesia, perineal conditions, being uplifted and hassled about pregnancy and satisfaction with the birth environment (Table 5).

**Table 5:** The results of linear regression analysis to investigate the effect of demographic and fertility characteristics, pregnancy experience and satisfaction with the birth environment on the birth experience in women (*n* = 225)

Independent variables		The statistics	Coefficient B	Standard factor	*p* Value^a^	*R* ^2^
Woman's education level	Elementary	0.895	1.493	0.089	.372	.398
	Guidance	1.173	1.773	0.104	.242	
	High school	−0.601	−0.785	−0.053	.549	
	Academic	Reference category				
Husband's education level	Elementary	0.373	0.655	0.036	.71	
	Guidance	−0.974	−1.415	−0.083	.331	
	High school	−2.044	−2.549	−0.175	**.042**	
	Academic	Reference category				
Getting regular care during pregnancy	Yes	−2.55	−5.898	−0.166	**.011**	
	No	Reference category				
The person giving birth	Gynaecologist	−1.062	−2.805	−0.09	.289	
	Midwife	2.602	6.308	0.194	**<.001**	
	Both	Reference category				
The presence of a companion during labour	Yes	2.642	2.55	0.912	**.002**	
	No	Reference category				
Type of delivery	Physiologic	1.028	1.167	0.072	.305	
	Intervention	Reference category				
Getting epidural or spinal anaesthetic	Yes	2.906	6.631	0.215	**.001**	
	No	Reference category				
Perineum status	Episiotomy	−0.297	−0.529	−0.022	.767	
	First or second degree tear	−2.547	−2.677	−0.177	**.012**	
	No laceration	Reference category				
Pregnancy experience (Hassle)		−0.537	−0.343	−0.272	**<.001**	
Pregnancy experience (Uplift)		3.28	0.538	0.86	**<.001**	
Satisfaction with the delivery environment		1.24	0.107	0.218	**.001**	

^a^ Significance level: *p* < .05 and the bold *p* values consider significant.

## 4 DISCUSSION

The results of the present study indicated the positive experience of childbirth as the score of childbirth experience was higher than the median score of the tool (52.5 of 100). In the study of Ghobadi, Ziaee, Mirhaghjo, and Pazandeh (2018), the score of childbirth experience (59.03) was also higher than the median score of the tool. In their study, only 7% of the women had university degrees, while in the current study 23% of women had university degrees (Ghobadi et al., 2018), women with lower levels of education were more satisfied with childbirth (Oweis, 2009). The results of a study in Jordan showed the low score of childbirth experience and 82.2% of the participants were dissatisfied with the care received during delivery. The lack of a formulated care plan for women at the time of delivery and ignore the emotional needs of them are two of the reasons for the dissatisfaction of women with the childbirth experience (Mohammad, Kassab, Gamble, Creedy, & Foster, 2014) (Mohammad et al., 2014).

Ghobadi et al. (2018) also showed that the participation of women in the labour and delivery process is another factor that affects the satisfaction of mothers with childbirth experience (Ghobadi et al., 2018). This result is consistent with the findings of the present study regarding the sense of participation. According to the study of Oweis, the mean score of women's perception of control during labour was 81.8 (58–107), which is not consistent with our findings, especially regarding the sense of control and security as one of the aspects of childbirth (Oweis, 2009).

Among the predictors of being uplifted and hassled in pregnancy experience, the mean score of the being uplifted was 24.41 ± 4.53 and being hassles was 18.67 ± 5.87. In the study conducted by DiPietro (2004), the mean scores of being uplifted and hassled domains were 20.75 and 13.14, respectively (DiPietro et al., 2004). The lower scores of being uplifted and hassled in the study of DiPietro in comparison with the current study can be related to the differences in the sampling time. The sampling in DiPietro's study was done during the 32nd to 36th weeks of pregnancy and before the start of delivery process, while in the present study sampling was carried out right after the beginning of delivery process and active phase. This can be a reason for the report of a higher level of hassle that is consistent with the report of Bettegowda et al. (2008) who suggested the increase in hassles of pregnant women when approaching the delivery time (Bettegowda et al., 2008). Also, the subjects in the study of DiPietro were all nulliparous, while only 20% of the participants in the current study were nulliparous and this can justify the higher level of hassle in the present study. According to the results of Saisto's study, women with previous experience of childbirth had more hassles during their recent pregnancy (Saisto, Ylikorkala, & Halmesmäki, 1999).

Among other predictors, the score of satisfaction with the birth environment was 80.65 ± 14.24, which was higher than the median score of the tool (50). Also, more than half of the women (61.3%) were very satisfied with the birth environment. The results of Mohammad et al. (2014) study showed that only 18.8% of the participants were satisfied with their birth environment (Mohammad et al., 2014).

Based on the results of the present study, the score of childbirth experience increased with the decreased sense of hassle and increased sense of uplift. The results of the present study are in line with the study of Jayasvasti and Kanchanatawan in Thailand that was conducted among 438 pregnant women (Jayasvasti & Kanchanatawan, 2005).

Our results also showed that the score of childbirth experience increased along with the increase in satisfaction with the birth environment. The results of Dolatian's study are consistent with the findings of the present study (Dolatian et al., 2008). The analysis in the study conducted by Pirdel and Pirdel showed that the environmental factors are the strongest predictors of labour pain (Pirdel & Pirdel, 2009).

The findings of the present study showed no significant relationship between the variables of age, occupation status, husband's age, husband's occupation status, place of residence, economic conditions, insurance, satisfaction with marital life and childbirth experience. However, there was a statistically significant relationship between the couples’ education and childbirth experience, so that the mean score of childbirth experience among women with high school education was significantly less than women who had elementary school education (*p* = .007) and secondary school education (*p* = .023). The findings of this research regarding the education of women are consistent with the results of a study conducted by Oweis (2009), which showed a relationship between childbirth experiences and women's education (Oweis, 2009). However, these results are not in line with the results of Jafari et al. and Dolatian et al. studies that showed the childbirth satisfaction increased along with the increase in mothers' education (Dolatian et al., 2008; Jafari, Mohebbi, & Mazloomzadeh, 2017).

Concerning the husband's education, our findings are consistent with the results of a study conducted by Peighambardost and Fadaiy (2016). Moreover, the study conducted by Ghobadi et al. (2018) found no significant relationship between the demographic characteristics of age, mother's occupation, family's income and childbirth experience.

The findings of the present study about the relationship between the fertility characteristics with childbirth experience showed that the mean score of childbirth experience was significantly higher in women who had not received regular pregnancy care, were accompanied by someone, gave physiologic birth and received epidural or spinal anaesthesia. Also, the mean score of childbirth experience among women who gave birth by a midwife was significantly higher than those who gave birth by a midwife and a gynaecologist. The mean score of childbirth experience in women with healthy perineum was significantly higher than those with first and second degree perineal lacerations (*p* = .032).

Our results are inconsistent with the findings of Mirmolaei's (2007) study in terms of receiving pregnancy care, as in the Mirmolaei's study childbirth satisfaction increased along with the increase in care received by mothers (Mirmolaei et al., 2007).

The results of the present study concerning the presence of a companion during labour are consistent with those of Nobakht, Rafiee Vardanjani, & Safdari Dahcheshmeh, (2012); Nouri, Afshari, Montazeri, & Latifi, (2008). The findings of the present study on the positive and significant relationship between physiologic birth and childbirth experience are consistent with the results of Jafari et al. study (Jafari, Mohebbi, Rastegari, & Mazloomzadeh, 2013).

Consistent with the results of the current study, Bakhshi, Taghizadeh, Amiri, and Kazemnagad (2015) showed that the mean score of childbirth experience among the women who received spinal anaesthesia was significantly higher than those who did not (Bakhshi et al.,^2015^).

The results of the present study on the relationship between the midwives being the people performing the birth are in line with the results of a study conducted by Pazandeh et al. that emphasized on the promotion of birth environments through appointing midwives as the directors of pregnancy care during labour and natural childbirth and also the supportive role of midwives that provide respectful evidence‐based care, improve childbirth care and play a positive role in improving the mothers' childbirth experience (Pazandeh, Potrata, Huss, Hirst, & House, 2017).

In the present study, no significant relationship was observed between the variables of the planned pregnancy, mother's parity and childbirth experience. This is while the study of Jafari et al. (2013) showed a significant relationship between planned pregnancy in the common delivery group, mother's parity in the physiologic birth and childbirth satisfaction (Jafari et al., 2013). The study of Dolatian et al. showed no significant relationship between the number of pregnancies, number of births, number of alive and stillborn children, history of abortion, infant's sex, wanted pregnancy and childbirth satisfaction. However, the above study showed a significant relationship between the age of the pregnancy, infant's sex matching the wishes of parents and childbirth satisfaction (Dolatian et al., 2008), but this relationship was not observed in the present study.

In the reporting of results based on the regression model, husband's education, receiving regular pregnancy care, the person giving birth, presence of a companion, receiving epidural or spinal anaesthesia, perineal conditions, being uplifted and hassled about pregnancy and satisfaction with the birth environment were some of the predictors of the childbirth experience. The regression model showed 39.8% of the changes in the outcome variable, which was the women's childbirth experience, could be predicted by the independent variables.

One of the limitations of the present study was the assessment period of pregnancy experience that was carried out during the labour process when pregnancy pain can influence the sense of being uplifted and hassled. It is recommended as a suggestion for future study that this questionnaire should be completed during the pregnancy period and before the beginning of the labour process. Another suggestion is to compare the childbirth experience of women giving birth physiologically with those who give birth using the intervention method, to compare the pregnancy experiences among the nulliparous and multiparous women and its relationship with the childbirth experience. This can help to examine the factors influencing the sense of women's participation in the labour and delivery process and design the interventions required to increase women's participation in the labour and delivery process.

## 5 CONCLUSION

In the present study, There was a positive relationship between the domains of the pregnancy experience, satisfaction with the birth environment and childbirth experience. According to the regression model, the variables of receiving regular care during pregnancy, the person giving birth, presence of a companion, type of delivery, receiving epidural or spinal anaesthesia and perineal conditions were the predictors of the childbirth experience.

## ETHICS APPROVAL AND CONSENT FOR PARTICIPATION IN RESEARCH

The study protocol was approved by the Ethics Committee of Iran University of Medical Sciences, Tehran, Iran (IR.IUMS.REC.1397.356) in August 2018. Furthermore, informed written consent was obtained from the participants and the respondents were completely informed of the study purpose and procedures. In addition, they were assured about the confidentiality of their information.

## CONFLICT OF INTERESTS

There is no conflict of interest.

## AUTHORS' CONTRIBUTIONS

F.K.G and L.A.F designed the study. F.K.G, L.A.F and S.H analysed and interpreted the data. Moreover, F.K.G and L.A.F wrote and revised the paper.

## References

[nop2603-bib-0001] Abbaspoor, Z. , Moghaddam‐Banaem, L. , Ronaghi, S. , & Dencker, A. (2019). Translation and cultural adaptation of the Childbirth Experience Questionnaire (CEQ) in Iran. Iranian Journal of Nursing and Midwifery Research, 24(4), 296. 31333745 10.4103/ijnmr.IJNMR_103_18 PMC6621505

[nop2603-bib-0002] Askari, F. , Atarodi, A. , Torabi, S. , & Moshki, M. (2014). Exploring women’s personal experiences of giving birth in gonabad city: A qualitative study. Global Journal of Health Science, 6(5), 46 10.5539/gjhs.v6n5p46 25168980 PMC4825378

[nop2603-bib-0003] Avni‐Barron, O. , & Wiegartz, P. (2011). Issues in treating anxiety disorders in pregnancy. Psychiatric times, 28(1), 47–50.

[nop2603-bib-0004] Babanazari, L. , Kafi, S. M. (2007). Comparative study of mental health and its relative demographic factors in different periods of pregnancy.

[nop2603-bib-0005] Bakhshi, M. , Taghizadeh, Z. , Amiri, L. , & Kazemnagad, A. (2015). A comparative study of birth experience, with or without spinal analgesia in nulliparous women admitted to the maternity ward of the selected hospital, Hamadan University of Medical Sciences in 1393. Tehran University of Medical Sciences School of Nursing and Midwifery.

[nop2603-bib-0006] Bell, A. F. , & Andersson, E. (2016). The birth experience and women's postnatal depression: A systematic review. Midwifery, 39, 112–123. 10.1016/j.midw.2016.04.014 27321728

[nop2603-bib-0032] Bettegowda, V. R. , Dias, T. , Davidoff, M. J. , Damus, K. , Callaghan, W. M. , & Petrini, J. R. (2008). The relationship between cesarean delivery and gestational age among US singleton births. Clinics in Perinatology, 35(2), 309–323. 18456071 10.1016/j.clp.2008.03.002

[nop2603-bib-0007] Dencker, A. , Taft, C. , Bergqvist, L. , Lilja, H. , & Berg, M. (2010). Childbirth experience questionnaire (CEQ): Development and evaluation of a multidimensional instrument. BMC Pregnancy and Childbirth, 10(1), 81 10.1186/1471-2393-10-81 21143961 PMC3008689

[nop2603-bib-0008] DiPietro, J. A. , Ghera, M. M. , Costigan, K. , & Hawkins, M. (2004). Measuring the ups and downs of pregnancy stress. Journal of Psychosomatic Obstetrics & Gynecology, 25(3–4), 189–201. 10.1080/01674820400017830 15715018

[nop2603-bib-0009] Dolatian, M. , Sayyahi, F. , & Simbar, M. (2008). Satisfaction rate of normal vaginal delivery and its relative factors among childbearing women in “Mahdiye, Tehran” and “Shaheed Chamran, Boroujerd” hospitals, 1385. Pajoohandeh Journal, 13(3), 259–268.

[nop2603-bib-0010] Elvander, C. , Cnattingius, S. , & Kjerulff, K. H. (2013). Birth experience in women with low, intermediate or high levels of fear: Findings from the first baby study. Birth, 40(4), 289–296. 10.1111/birt.12065 24344710 PMC3868996

[nop2603-bib-0011] Fair, C. D. , & Morrison, T. E. (2012). The relationship between prenatal control, expectations, experienced control and birth satisfaction among primiparous women. Midwifery, 28(1), 39–44. 10.1016/j.midw.2010.10.013 21458895

[nop2603-bib-0012] Fenwick, J. , Staff, L. , Gamble, J. , Creedy, D. K. , & Bayes, S. (2010). Why do women request caesarean section in a normal, healthy first pregnancy? Midwifery, 26(4), 394–400. 10.1016/j.midw.2008.10.011 19117644

[nop2603-bib-0013] Ghobadi, M. , Ziaee, T. , Mirhaghjo, N. , & Pazandeh, F. (2018). Evaluation of satisfaction with natural delivery experience and its related factors in Rasht women. Journal of Health and Care, 20(3), 215–224. 10.29252/jhc.20.3.215

[nop2603-bib-0014] Hajifoghaha, M. , Abbas, E. , & Nourossadat, K. (2016). Persian translation of the pregnancy experience scale (PES)–brief version: Confirmatory factor analysis. Pakistan Journal of Medical & Health Sciences, 12(1), 503–508.

[nop2603-bib-0033] Jafari, E. , Mohebbi, P. , & Mazloomzadeh, S. (2017). Factors related to women's childbirth satisfaction in physiologic and routine childbirth groups. Iranian Journal of Nursing and Midwifery Research, 22(3), 219. 28706547 10.4103/1735-9066.208161 PMC5494952

[nop2603-bib-0015] Jafari, E. , Mohebbi, P. , Rastegari, L. , & Mazloomzadeh, S. (2013). The comparison of physiologic and routine method of delivery in mother’s satisfaction level in Ayatollah Mosavai Hospital, Zanjan, Iran, 2012. The Iranian Journal of Obstetrics, Gynecology and Infertility, 16(73), 9–18.

[nop2603-bib-0016] Jayasvasti, K. , & Kanchanatawan, B. (2005). Happiness and related factors in pregnant women. Journal of the Medical Association of Thailand=. Chotmaihet Thangphaet, 88, S220–225. 16623032

[nop2603-bib-0017] Larkin, P. , Begley, C. M. , & Devane, D. (2012). ‘Not enough people to look after you’: An exploration of women's experiences of childbirth in the Republic of Ireland. Midwifery, 28(1), 98–105. 10.1016/j.midw.2010.11.007 21237541

[nop2603-bib-0018] Larsson, C. , Saltvedt, S. , Edman, G. , Wiklund, I. , & Andolf, E. (2011). Factors independently related to a negative birth experience in first‐time mothers. Sexual & Reproductive Healthcare, 2(2), 83–89. 10.1016/j.srhc.2010.11.003 21439526

[nop2603-bib-0019] Lobel, M. , Cannella, D. L. , Graham, J. E. , DeVincent, C. , Schneider, J. , & Meyer, B. A. (2008). Pregnancy‐specific stress, prenatal health behaviors and birth outcomes. Health Psychology, 27(5), 604 10.1037/a0013242 18823187

[nop2603-bib-0020] McLachlan, H. L. , Forster, D. A. , Davey, M.‐A. , Farrell, T. , Gold, L. , Biro, M. A. , … Waldenström, U. (2012). Effects of continuity of care by a primary midwife (caseload midwifery) on caesarean section rates in women of low obstetric risk: The COSMOS randomised controlled trial. BJOG: An International Journal of Obstetrics & Gynaecology, 119(12), 1483–1492. 22830446 10.1111/j.1471-0528.2012.03446.x

[nop2603-bib-0021] Mirmolaei, S. , Khakbazan, Z. , Kazemnejad, A. , & Azari, M. (2007). Prenatal care utilization rate and patients satisfaction. Journal of Hayat, 13(2), 31–40.

[nop2603-bib-0022] Mohammad, K. , Kassab, M. , Gamble, J. , Creedy, D. K. , & Foster, J. (2014). Factors associated with birth weight inequalities in Jordan. International Nursing Review, 61(3), 435–440. 25081475 10.1111/inr.12120

[nop2603-bib-0023] Nasiry, F. , & Sharifi, S. (2013). Relationship between fear of childbirth and personality type in pregnant women. The Iranian Journal of Obstetrics, Gynecology and Infertility, 16(66), 18–25.

[nop2603-bib-0024] Nobakht, F. , Rafiee Vardanjani, L. , & Safdari Dahcheshmeh, F. (2012). The effect of the presence of an attendant on anxiety and labor pain of primiparae referring to Hajar Hospital in Shahre Kurd, 2010. Journal of Research Development in Nursing & Midwifery, 9(1), 41–50.

[nop2603-bib-0025] Nouri, M. , Afshari, P. , Montazeri, S. , & Latifi, S. (2008). The effect of continuous labor support by accompanying person during labor process.

[nop2603-bib-0026] Oweis, A. (2009). Jordanian mother's report of their childbirth experience: Findings from a questionnaire survey. International Journal of Nursing Practice, 15(6), 525–533. 19958407 10.1111/j.1440-172X.2009.01774.x

[nop2603-bib-0027] Oyira, E. J. , Mary, M. , Osuchukwu, E. C. , Onoyom, A. E. , Lukpata, F. E. , & Manyo, O.‐A.‐M. (2016). Delivery pain anxiety/fear control between midwives among women in Cross River State, Nigeria. Journal of Education and Training Studies, 4(3), 138–149.

[nop2603-bib-0031] Pazandeh, F. , Potrata, B. , Huss, R. , Hirst, J. , & House, A. (2017). Women’s experiences of routine care during labour and childbirth and the influence of medicalisation: a qualitative study from Iran. Midwifery, 53, 63–70. 28763721 10.1016/j.midw.2017.07.001

[nop2603-bib-0028] Peighambardost, R. , & Fadaiy, Z. (2016). Effect of Telephone support and Women satisfaction of Postpartum care. Journal of Clinical Nursing and Midwifery, 5(1), 36–46.

[nop2603-bib-0029] Pirdel, M. , & Pirdel, L. (2009). Perceived environmental stressors and pain perception during labor among primiparous and multiparous women. Journal of Reproduction & Infertility, 10(3), 217. 23926472 PMC3719331

[nop2603-bib-0035] Saisto, T. , Ylikorkala, O. , & Halmesmäki, E. (1999). Factors associated with fear of delivery in second pregnancies. Obstetrics & Gynecology, 94(5), 679–682. 10546709 10.1016/s0029-7844(99)00413-5

[nop2603-bib-0030] Toohill, J. , Fenwick, J. , Gamble, J. , Creedy, D. K. , Buist, A. , Turkstra, E. , & Ryding, E. L. (2014). A randomized controlled trial of a psycho‐education intervention by midwives in reducing childbirth fear in pregnant women. Birth, 41(4), 384–394. 25303111 10.1111/birt.12136 PMC4257571

